# High‐Performance Synapse Arrays for Neuromorphic Computing via Floating Gate‐Engineered IGZO Synaptic Transistors

**DOI:** 10.1002/advs.202500568

**Published:** 2025-03-20

**Authors:** Junhyeong Park, Yumin Yun, Sunyeol Bae, Yuseong Jang, Seungyoon Shin, Soo‐Yeon Lee

**Affiliations:** ^1^ Department of Electrical and Computer Engineering, and Inter‐university Semiconductor Research Center (ISRC) Seoul National University Seoul 08826 Republic of Korea

**Keywords:** InGaZnO (IGZO), neuromorphic computing, spiking neural network, synapse array, synaptic transistor

## Abstract

Neuromorphic computing emulating the human brain offers a promising alternative to the Von Neumann architecture. Developing artificial synapses is essential for implementing hardware neuromorphic systems. Indium‐gallium‐zinc oxide (IGZO)‐based synaptic transistors using charge trapping have advantages, such as low‐temperature process and complementary metal‐oxide‐semiconductor compatibility. However, these devices face challenges of low charge de‐trapping efficiency and insufficient retention. Here, IGZO synaptic transistors are introduced utilizing an indium‐tin oxide (ITO) floating gate (FG) to overcome these limitations. The ITO FG's higher conductivity and alleviated chemical interactions with the Al_2_O_3_ tunneling layer (TL) deposited by atomic layer deposition result in enhanced electrical performance with a smooth FG/TL interface. An 8 × 8 synapse array achieves 100% yield and successful programming without interference using a half‐pulse scheme. Spiking neural network simulations on MNIST and Fashion‐MNIST datasets demonstrate high accuracies of 98.31% and 87.76%, respectively, despite considering device variations and retention. These findings highlight the potential of IGZO synaptic transistors for neuromorphic computing applications.

## Graphical abstract

Indium‐gallium‐zinc oxide synaptic transistors are introduced utilizing an indium‐tin oxide (ITO) floating gate (FG). The ITO FG's higher conductivity and alleviated chemical interactions with the Al_2_O_3_ tunneling layer contribute to enhanced performance. An 8 × 8 synapse array achieves 100% yield, and spiking neural network simulations on MNIST and Fashion‐MNIST datasets demonstrate high accuracies of 98.31% and 87.76%, respectively.

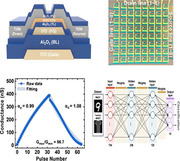

## 1 Introduction

With the advent of deep learning and machine learning, there is great interest in exploring new computing paradigms that can handle significant amounts of data efficiently with minimization of data movement between storage and computation units.^[^ ^1^, ^2^ ^]^ Recently, neuromorphic computing, inspired by the human brain that can perform diverse functionalities with only 20 watts, has been attracting attention to overcome the inefficiency stemming from the Von Neumann computing architecture.^[^ ^3^, ^4^ ^]^ This computing technology utilizes parallel‐connected artificial neurons and synapses, where synapses function as storing the information by adjusting the strength of the connection between neurons for realizing learning and data processing.^[^ ^5^ ^]^ To advance toward the implementation of hardware‐based neuromorphic computing, intensive efforts have been made to develop artificial synapses that can emulate synaptic plasticity using materials such as silicon,^[^ ^6^, ^7^ ^]^ metal oxide,^[^ ^8^, ^9^ ^]^ and 2D semiconductors.^[^ ^10^, ^11^ ^]^ Notably, indium‐gallium‐zinc oxide (IGZO) is a promising metal oxide material for artificial synapses due to its advantages of low leakage current, low processing temperature, and ease of deposition.^[^ ^12^, ^13^, ^14^, ^15^ ^]^ Many studies on IGZO‐based synaptic transistors have been conducted, utilizing ferroelectric material,^[^ ^16^, ^17^ ^]^ electrical double layer,^[^ ^18^, ^19^ ^]^ and charge trapping layer.^[^ ^20^, ^21^ ^]^ IGZO synaptic transistors based on charge trapping offer the benefits of complementary metal‐oxide‐semiconductor compatibility, linear weight modulation, and stable weight updates.^[^ ^22^, ^23^, ^24^ ^]^ However, this type of device has an intrinsic problem originating from the n‐type IGZO channel, which has low charge de‐trapping efficiency because it is difficult to supply holes from the channel during the charge de‐trapping process.^[^ ^25^, ^26^ ^]^ Alternatively, previous studies have used light irradiation on the channel to temporarily generate electron‐hole pairs to improve the charge de‐trapping efficiency.^[^ ^27^, ^28^, ^29^ ^]^

To address this issue, our group employed highly conductive degenerate IGZO as a floating gate (FG) and successfully implemented the synaptic plasticity with enhanced charge de‐trapping efficiency without the need for light irradiation.^[^ ^30^ ^]^ However, despite the use of the IGZO FG, there are still remaining challenges to be addressed to meet the requirements for an artificial synapse: insufficient retention characteristics of synaptic weights and the requirement for high programming voltage for potentiation and depression (≈20 V).^[^ ^31^, ^32^ ^]^ These issues are primarily related to the tunneling layer (TL) where charge transport occurs, and securing a defect‐less FG/TL interface is crucial for preventing charge loss and degradation of tunneling efficiency.^[^ ^33^, ^34^, ^35^ ^]^ However, it is known that the Al_2_O_3_ layer, which is commonly used as a TL, can induce undesirable chemical reactions with IGZO during the atomic layer deposition (ALD) process, forming a defect‐rich region reacting with the surface of IGZO.^[^ ^36^, ^37^ ^]^ The trimethylaluminum (TMA) precursor used in the ALD process of Al_2_O_3_, which acts as a strong reducing agent, is the main cause for defect‐rich interfaces for metal oxides such as IGZO,^[^ ^38^ ^]^ In_2_O_3_,^[^ ^39^, ^40^ ^]^ and ZnO.^[^ ^41^ ^]^ Therefore, attention should be paid to suppressing this redox reaction with the ALD‐deposited Al_2_O_3_ layer, as the reaction can degrade the FG/TL interface. Furthermore, since the conductivity of FG determines the charge de‐trapping efficiency,^[^ ^30^, ^36^ ^]^ adopting highly conductive FG is important to further reduce the programming voltages. Therefore, it is necessary to engineer an FG that can achieve high conductivity while minimizing chemical reactions with ALD‐deposited films, overcoming the limitations of the IGZO synaptic transistor.

In this paper, we fabricated a high‐performance synaptic transistor by introducing an indium‐tin oxide (ITO) FG that shows superior program/erase and retention characteristics compared to the IGZO FG. Various electrical and chemical analyses were conducted to investigate the cause of performance differences between ITO and IGZO FGs. The ITO FG exhibited less reactivity with ALD‐deposited Al_2_O_3_, resulting in smoother interface and reduced cation diffusions verified by transmission electron microscopy (TEM) and X‐ray photoelectron spectroscopy (XPS) analyses. It was found that the crystalline nature of ITO contributes to the suppression of redox reactions for TMA precursor, whereas amorphous IGZO shows the formation of a defect‐rich layer near the interface. Using the ITO FG synaptic transistor, synaptic characteristics such as long‐term potentiation (LTP) and long‐term depression (LTD) were measured, showing the capability of linear conductance modulation and multi‐state retention. In addition, an 8 x 8 synapse array was fabricated to verify the array operation, exhibiting a high yield of 100% with low device‐to‐device variation. Weight programming in the array was successfully performed using the half‐pulse scheme on the target cell without interference with adjacent cells. Based on the measured data, simulations of spiking neural network (SNN) systems were conducted using the MNIST and Fashion‐MNIST datasets. The system showed high accuracies of 98.31% and 87.76% for the MNIST and Fashion‐MNIST, respectively, even when considering device variation and retention characteristics.

## 2 Results and Discussion

**Figure 1:**
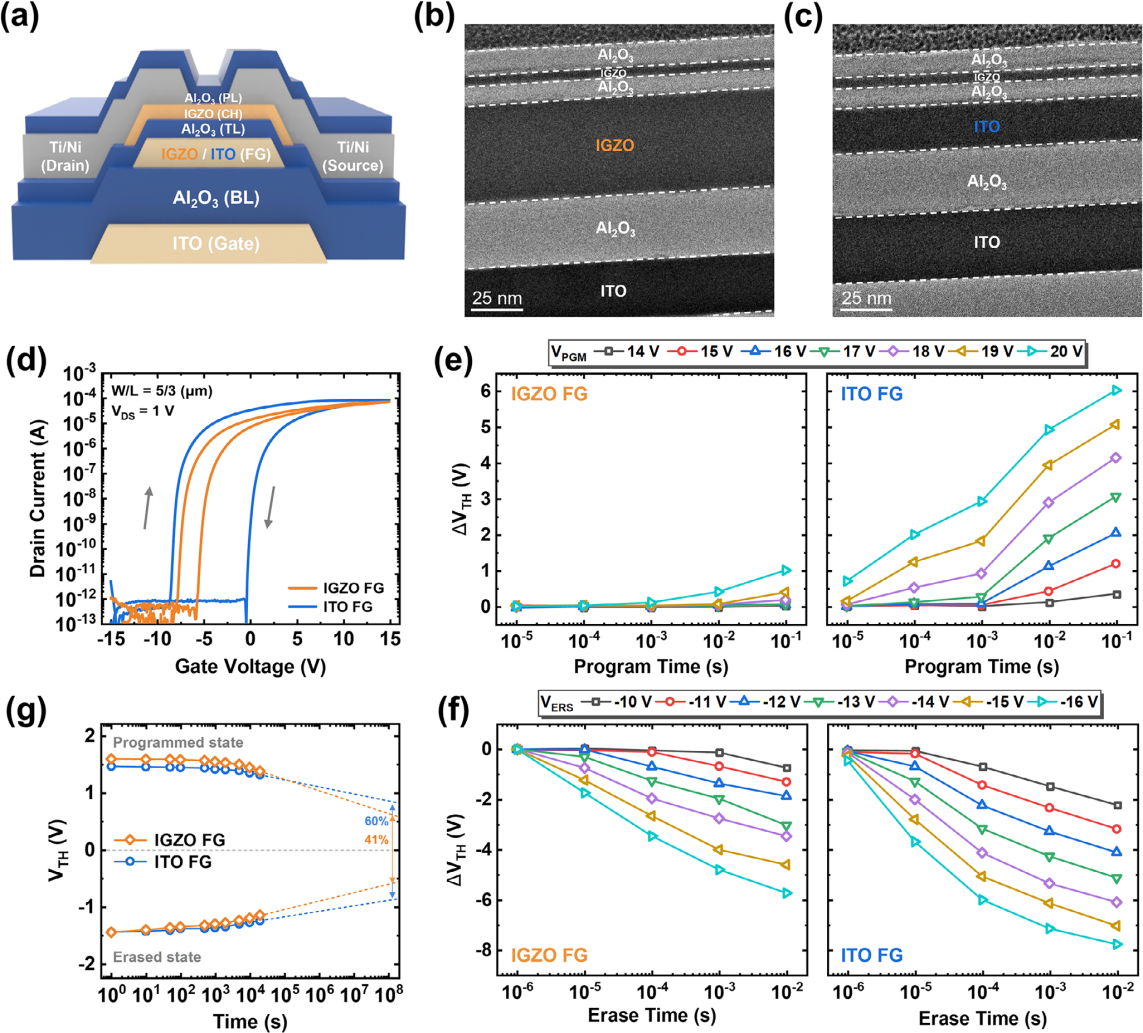
a) Schematic of the synaptic transistors with IGZO and ITO FGs, respectively. Cross‐sectional TEM images of the synaptic transistors with b) IGZO FG and c) ITO FG. d) Transfer curves of synaptic transistors under gate double‐sweep (±15 V) according to FG material. Comparison of ΔV_TH_ with various pulse widths and magnitudes for e) program and f) erase operations. g) Retention characteristics of programmed and erased states for 2 x 10^4^ s for the IGZO and ITO FGs.

We adopted ITO as an alternative to IGZO FG to fabricate a synaptic transistor driven by two primary motivations. First, ITO is known as an n‐type degenerate semiconductor that can achieve a high carrier concentration through the doping of Sn acting as a cationic dopant.^[^ ^42^ ^]^ Therefore, it can attain higher conductivity with a thinner film than IGZO, and our experimental results confirmed that ITO exhibits higher conductivity than IGZO (Figure S1, Supporting Information). Given that higher conductivity enhances the efficiency of charge de‐trapping, employing ITO can be beneficial in lowering the programming voltage.^[^ ^36^ ^]^ Second, the crystallinity of ITO can be tailored through deposition conditions,^[^ ^43^ ^]^ which may mitigate reactions with the TMA precursor because previous study reported that enhanced crystallization in In_2_O_3_ can suppress chemical reactions with the ALD‐deposited Al_2_O_3_ layer.^[^ ^40^ ^]^ Likewise, crystallized ITO is expected to exhibit a similar effect, minimizing reactions with the Al_2_O_3_ TL. Motivated by these findings, we fabricated synaptic transistors using an ITO FG, expecting superior performance compared to those with an IGZO FG. **Figure** **1**a presents the cross‐sectional structure of synaptic transistors with IGZO and ITO FGs. When a high voltage is applied to the gate terminal, threshold voltage (V_TH_) can be modulated through electron transfer between the channel and FG via Fowler‐Nordheim (FN) tunneling, which is enabled by the substantial voltage drop across the TL and the negligible voltage drop within the FG.^[^ ^30^ ^]^ It is noteworthy that a synaptic transistor with the IGZO FG was also fabricated to evaluate the impact of the FG material on device performance. Figure 1b,c shows cross‐sectional TEM images of devices with ITO and IGZO FGs, respectively, where each layer is clearly distinguishable. Energy‐dispersive spectroscopy (EDS) analysis mapping results confirm that all the layers are well‐deposited and correspond to their respective elements (Figure S2, Supporting Information). To compare the electrical properties of the fabricated devices, transfer curves were measured under a gate double sweep from −15 to 15 V, where the clear clockwise hysteresis was observed for both devices with IGZO and ITO FGs. Since hysteresis can result from the interface trapping at the channel/TL interface rather than from charge transfer between channel and FG, a switching transistor without an FG was fabricated under the same process conditions and measured with the same gate double‐sweep (Figure S3, Supporting Information). The switching transistor exhibited negligible hysteresis, suggesting that charge trapping/de‐trapping occur in FG. The device with the ITO FG shows a larger hysteresis window of 8.2 V compared to 2.1 V for the IGZO FG, implying that the ITO FG significantly enhances charge trapping and de‐trapping behaviors. In addition, the device can operate in both depletion and enhancement modes according to the gate voltage conditions (Figure S4, Supporting Information). To further analyze charge trapping/de‐trapping behaviors, program and erase operations were performed using various pulse widths and magnitudes (Figure 1e,f). The IGZO FG exhibited lower threshold voltage shifts (ΔV_TH_) in both program and erase operations than the ITO FG. As the conductivity of FG is critical for charge de‐trapping efficiency, it is reasonable to infer that IGZO, which has lower conductivity than ITO, exhibits less ΔV_TH_ in erase operation (i.e., charge de‐trapping) compared to ITO.^[^ ^30^, ^36^ ^]^ Nevertheless, it should be noted that ΔV_TH_ is significantly reduced in the program operation for the IGZO FG. Under a 20V/100 ms voltage pulse, the device with IGZO FG exhibits a lower ΔV_TH_ of 1 V compared to 6 V for the ITO FG, indicating that IGZO FG experiences abnormal degradation in the charge trapping. Additionally, when evaluating the retention characteristics in both programmed and erased states, the device with ITO FG exhibited a slower decline in V_TH_ margin (V_TH_programmed_ ‐V_TH_erased_) compared to the IGZO FG device. The retention measurement of programmed and erased states was conducted using a similar ΔV_TH_ from initial V_TH_ in the devices with ITO and IGZO FGs (Figure S5, Supporting Information). After interpolation to 10^8^ seconds, the ITO FG retained 60% of the initial V_TH_ margin, whereas the IGZO FG retained only 41%, showing a faster margin reduction. Given that charge tunneling and leakage primarily occur at the TL, the degradation in program and retention characteristics in the IGZO FG could be attributed to a deterioration of the interface quality between the FG and TL.

**Figure 2:**
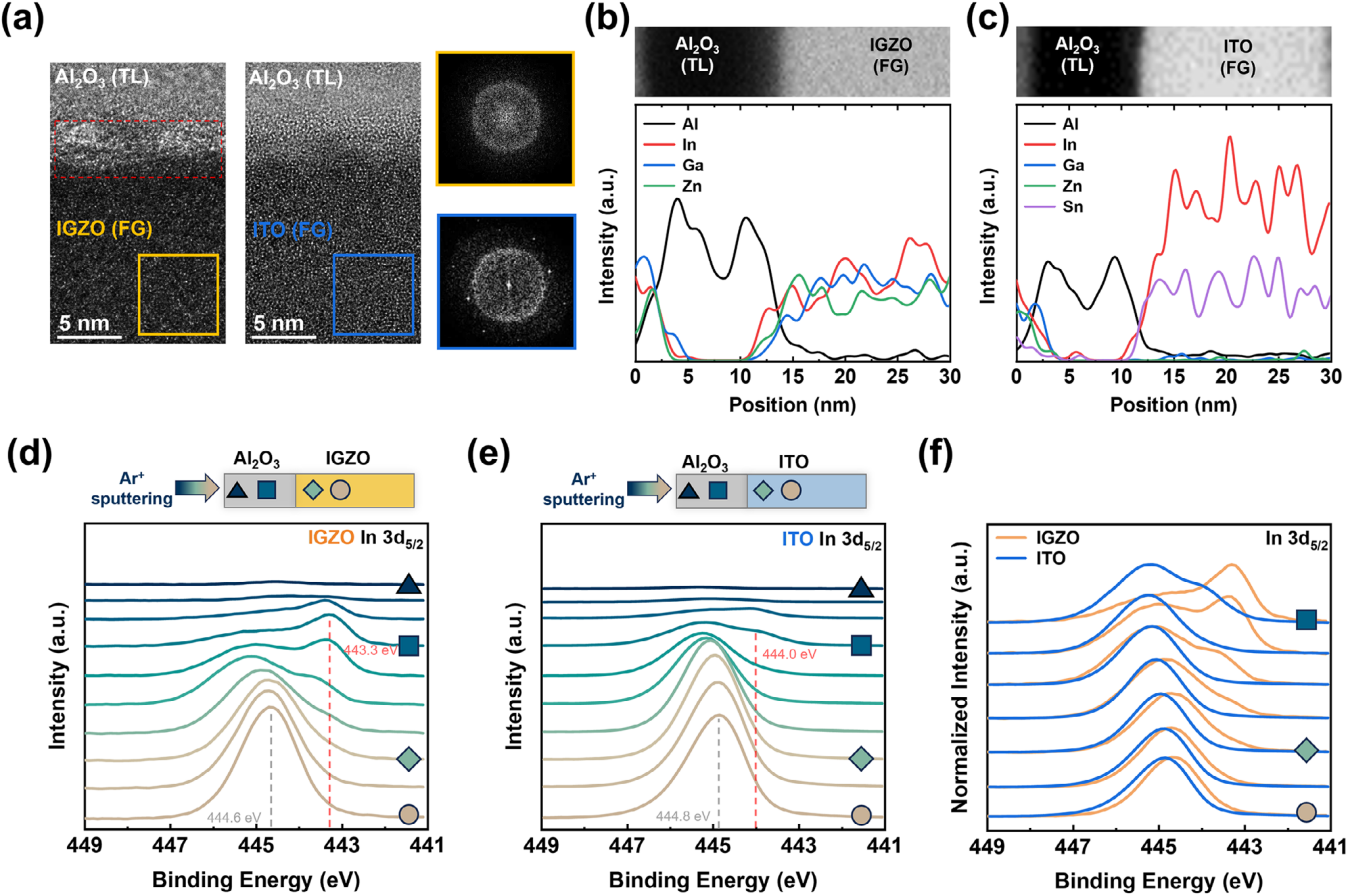
a) Cross‐sectional TEM images of TL/FG interface region (left) and corresponding FFT images (right) for IGZO and ITO FGs. EDS line scan profiles for b) Al_2_O_3_/IGZO and c) Al_2_O_3_/ITO regions. Depth‐resolved XPS core level spectra of d) Al_2_O_3_/IGZO and e) Al_2_O_3_/ITO stacks using Ar^+^ sputtering. f) Normalized In 3d_5/2_ spectra according to FG materials near the interface.

To investigate the underlying reasons for differences in the device performance with respect to FG material, we first analyzed the TL/FG interface of the synaptic transistors using TEM. The analysis revealed a rough IGZO/Al_2_O_3_ interface in contrast to a smooth ITO/Al_2_O_3_ interface, as shown in **Figure** **2**a. EDS line scan analysis at the FG/TL interface was performed to identify the elements contributing to the formation of the rough interface (Figure 2b,c). For the IGZO FG, In diffusion from IGZO into Al_2_O_3_ was observed, consistent with the interdiffusion caused by the chemical reaction between IGZO and the TMA precursor in the Al_2_O_3_ ALD process.^[^ ^38^ ^]^ In contrast, there was no distinguishable diffusion of In or Sn into the Al_2_O_3_ for the ITO FG. To further investigate the chemical states and composition at the FG/TL interface, which are crucial for understanding the mechanism of interface degradation, XPS depth profiling was conducted. Figure 2d,e presents the depth‐resolved XPS core level spectra of In 3d_5/2_ near the interface for Al_2_O_3_/ITO and Al_2_O_3_/IGZO stacks. For the Al_2_O_3_/IGZO stack, a pronounced shoulder peak at lower binding energy (BE) was observed as the analysis approached the interface. The BE of the shoulder peak is 443.3 eV, which is 1.3 eV lower than the bulk peak at 444.6 eV. This BE shift indicates a change in the binding state of In near the interface, suggesting that the reduction of In ions results in mixed states of In^2+^, In^1+^, and In^0^.^[^ ^40^, ^44^ ^]^ In contrast, regarding the Al_2_O_3_/ITO stack, a shoulder peak was observed in In 3d_5/2_ spectra but with lower intensity and a smaller peak shift compared to the Al_2_O_3_/IGZO stack, where the BE of the shoulder and bulk peak are 444.0 and 444.8 eV, respectively. Noticeable shoulder peaks or peak shifts were not observed for other elements (Ga, Zn, and Sn) at the Al_2_O_3_/IGZO or Al_2_O_3_/ITO interface (Figure S6, Supporting Information) because In has the lowest negative Gibbs free energy for reaction with TMA precursor, making the redox reaction of In thermodynamically favored.^[^ ^45^ ^]^ Normalized In 3d_5/2_ core level spectra near the interface exhibited apparent differences in shoulder peak shape and BE between Al_2_O_3_/ITO and Al_2_O_3_/IGZO stacks, indicating that the change in the chemical state of In is alleviated when using ITO (Figure 2f). In addition, low‐frequency noise measurements were performed to evaluate the interface quality of ITO and IGZO FGs (Figure S7, Supporting Information). The device with IGZO FG exhibited a higher normalized noise spectral density than the device with ITO FG, providing additional evidence of degraded interface quality in the IGZO FG. Subsequently, TEM fast Fourier transform (FFT) images (Figure 2a) and grazing incidence X‐ray diffraction (GIXRD) analysis (Figure S8, Supporting Information) were used to evaluate the crystallinity of FG, revealing that ITO exhibits a polycrystalline structure, whereas IGZO is amorphous. Considering the fact that the interactions with ALD‐deposited Al_2_O_3_ diminish as the crystallinity of InO enhances,^[^ ^40^ ^]^ it is deduced that the crystallization of ITO contributes to the mitigation of redox reactions with Al_2_O_3_ TL compared to IGZO, thereby achieving an FG/TL interface with reduced defects of interstitial In.

**Figure 3:**
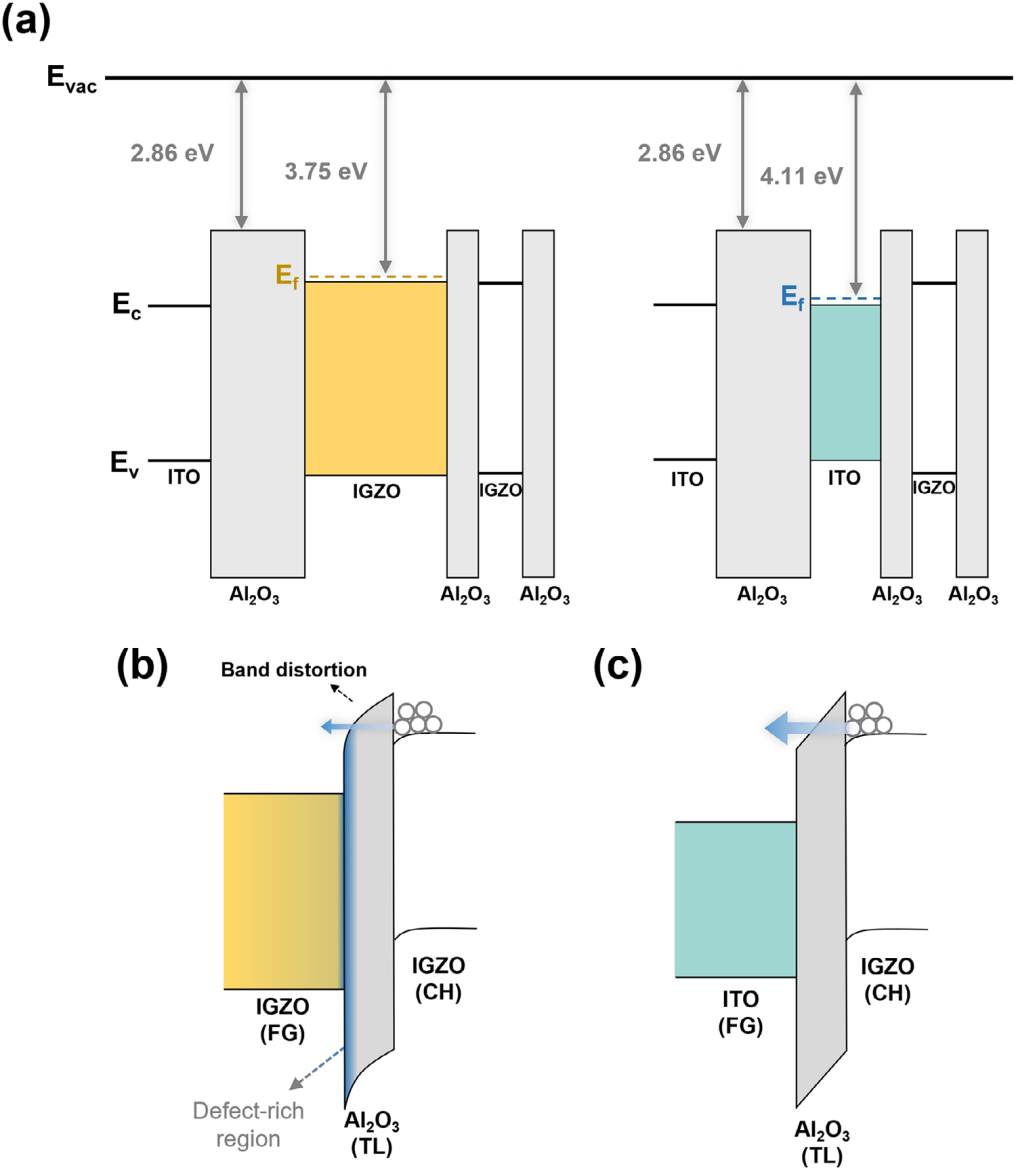
a) Schematic energy band diagrams of the synaptic transistors with IGZO and ITO FGs. Program operations of the devices with b) IGZO and c) ITO FGs, illustrating the differences in tunneling barriers.

To determine the band structures of each material layer, several analysis methods of reflective electron energy loss spectroscopy (REELS), ultraviolet photoelectron spectroscopy (UPS), and ultraviolet‐visible (UV‐vis) spectroscopy were performed (Figure S9, Supporting Information). **Figure** **3**a illustrates the flat band structure of the synaptic transistors with ITO and IGZO FGs, respectively. The analysis results demonstrate that both IGZO and ITO FGs are degenerate, with Fermi level (E_f_) positions above the conduction band (E_c_). Specifically, E_f_ is 0.04 eV above E_c_ for IGZO and 0.13 eV above E_c_ for ITO, consistent with the low resistance of IGZO and ITO (Figure S1, Supporting Information). Figure 3b,c presents the energy band diagram according to the FG when a high positive gate voltage is applied to induce the FN tunneling between FG and TL. As previously discussed, the program operation in the IGZO FG was significantly degraded, and the interface analysis revealed the presence of defect‐rich regions, which can serve as trap sites during electron tunneling. Electrons trapped in these defects can cause band distortion, subsequently reducing the electric field and decreasing the number of tunneled electrons via FN tunneling.^[^ ^46^, ^47^, ^48^ ^]^ Consequently, the charge trapping efficiency is substantially diminished, correlating with a large decrease of ΔV_TH_ during the program operation. In contrast, the ITO FG facilitates more efficient electron tunneling through the TL with high quality FG/TL interface. Furthermore, ITO FG exhibited improved retention characteristics compared to the IGZO FG because charge leakage, possibly caused by direct tunneling and trap‐assisted tunneling,^[^ ^34^, ^49^ ^]^ was mitigated due to the decreased defects in Al_2_O_3_ TL. In addition, the higher electron affinity of ITO compared to IGZO could contribute to improved retention at high temperatures by providing a larger energy barrier between the FG and TL because charge loss can occur not only via tunneling but also via thermal emission at high temperatures.^[^ ^49^, ^50^, ^51^ ^]^

**Figure 4:**
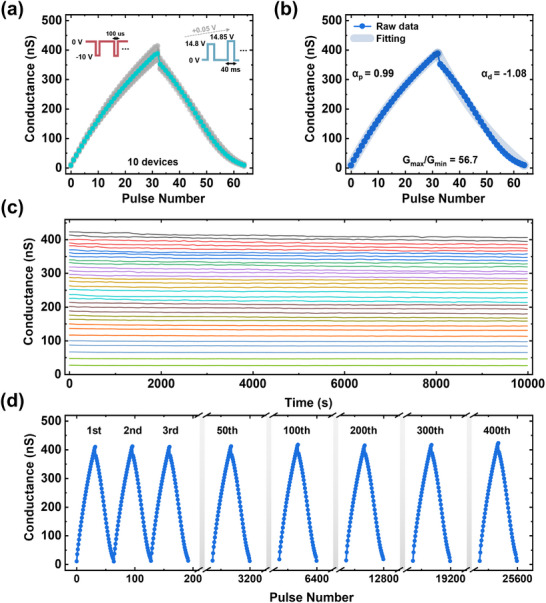
a) LTP/LTD measurement results for 10 individual synaptic transistors. b) Nonlinearity fitting results of average LTP/LTD values from 10 devices. c) Retention characteristics of the synaptic transistor, exhibiting distinct multi‐states for 10^4^ s. d) Repetitive LTP/LTD measurement results of the synaptic transistor using 25 600 pulses.

Since the ITO FG synaptic transistor exhibited superior program/erase and retention characteristics, LTP and LTD measurements were performed on this device, as these properties are critical for achieving high neural network accuracy.^[^ ^52^ ^]^ To assess the capability of conductance modulation, consecutive voltage pulses were applied to the gate terminal: −10 V/100 µs for potentiation and 14.8 to 16.35 V/40 ms for depression. **Figure** **4**a presents the LTP/LTD results for 10 individual devices, demonstrating clear and uniform conductance modulation using 32 pulses for both LTP and LTD. An incremental pulse scheme was employed to enhance the linearity of LTD because an identical pulse scheme degraded the linearity of LTD (Figure S10, Supporting Information). In addition, the synaptic transistor exhibits linear weight modulation using the same voltage pulse width for both LTP and LTD (Figure S11, Supporting Information). Although the incremental pulse scheme can enhance the linearity of synaptic transistors, it also complicates the programming process, increasing the complexity of peripheral circuit. Therefore, an appropriate pulse scheme should be selected by considering the trade‐off between linearity and system implementation. For a quantitative evaluation of the linearity, nonlinearity parameters of α_p_ and α_d_ were fitted using the equations:^[^ ^53^ ^]^

(1) $$G_{p}=B(1-e^{-P/A_{p}})+G_{\min}$$

(2) $$G_{d}=-B(1-e^{(P-P_{\max})/A_{d}})+G_{\max}$$

(3) $$B\;=\frac{\left(G_{\max}-G_{\min}\right)}{\left(1-e^{-P_{\max}/A_{p,d}}\right)}$$

where G_P_, G_d_, P, and P_max_ denote the conductance of potentiation, the conductance of depression, the number of pulses, and the maximum pulse number, respectively. The parameters A_p_ and A_d_ represent the fitting parameters for the nonlinearity of potentiation and depression. The nonlinearity factors α_p_ and α_d_ are subsequently obtained from a predefined table using A_p_ and A_d._ Figure 4b demonstrates the fitting results of the average LTP/LTD values from 10 devices, where the α_p_ and α_d_ are 0.99 and −1.08, respectively. The synaptic transistor shows linear multi‐state conductance modulation with a high dynamic range (G_max_/G_min_) of 56.7. Besides these synaptic characteristics, retention plays an important role in determining neural network accuracy, and poor retention can lead to increased system energy consumption due to frequent weight refresh operations.^[^ ^54^, ^55^ ^]^ Figure 4c presents the retention measurements of multi‐state for 10^4^ s, showing that each state remains stable and distinguishable, with an average conductance change of only 3.77% after 10^4^ s. Compared to the IGZO FG device, the ITO FG synaptic transistor shows superior retention characteristics at the same conductance state due to the robustness of the ITO FG against chemical reactions with ALD‐deposited TL (Figure S12, Supporting Information). Endurance, as an essential property for reliable neuromorphic systems,^[^ ^56^ ^]^ was also evaluated using 25600 pulses to implement repetitive LTP/LTD behaviors, as shown in Figure 4d. In addition, the endurance of repetitive program and erase operations was evaluated using larger voltage pulses than those used for LTP and LTD measurements (Figure S13, Supporting Information). The synaptic transistor exhibits robust endurance, with no degradation in the conductance range.

**Figure 5:**
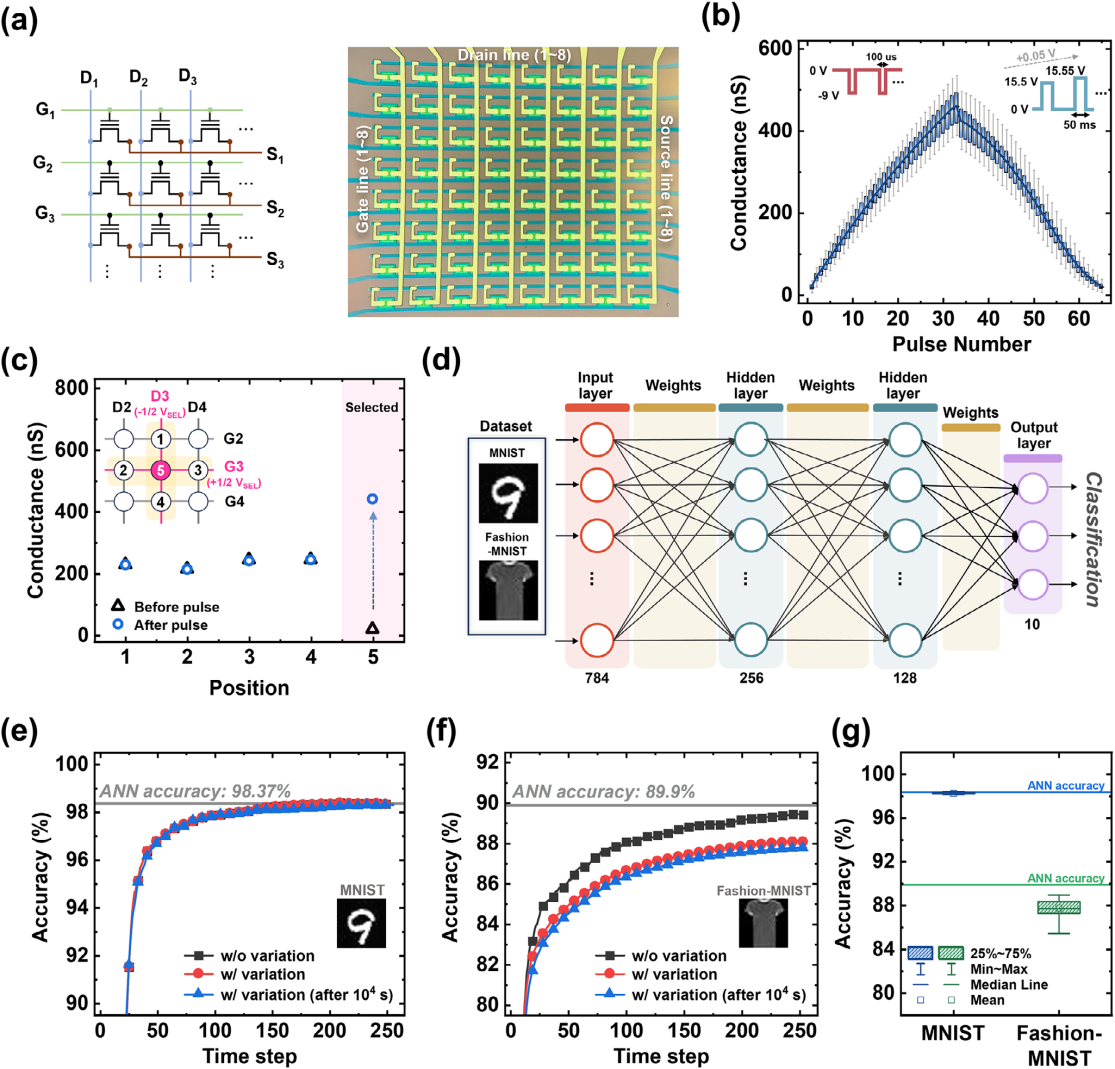
a) Schematic of the synaptic transistor array (left) and optical image of the fabricated ITO FG synaptic transistor array (right). b) LTP/LTD measurement results of all 64 devices in the array. c) Conductance changes of the selected device and adjacent devices after applying the pulse, with the pulse scheme used for updating the synaptic weight at the selected device (inset). d) Schematic of the network structure for SNN simulation with MNIST and Fashion‐MNIST datasets. SNN accuracy results as a function of the time step for e) MNIST and f) Fashion‐MNIST datasets, considering device‐to‐device variation and retention characteristics. g) Box plot of accuracy results from 20 simulations for the MNIST and Fashion‐MNIST datasets.

We fabricated an 8x8 NOR‐type synapse array using ITO FG synaptic transistors to verify the array operation, as depicted in **Figure** **5**a. Transfer curves for all devices in the array were measured, confirming a 100% yield, with clear clockwise hysteresis observed (Figure S14, Supporting Information). To investigate device‐to‐device variation in LTP/LTD performance, a series of LTP/LTD pulses were applied to all 64 devices, which resulted in a low variation (σ/μ) of 12.5% (Figure 5b). In synaptic transistor arrays, programming a single device can cause interference in other devices sharing the same gate line, possibly reducing neural network accuracy.^[^ ^57^ ^]^ To address this issue, we implemented a half‐pulse scheme that enables FN tunneling to occur in the selected device. Instead of applying the full programming voltage V_SEL_ to the gate line, the half‐pulse scheme applies +1/2V_SEL_ to the gate and −1/2V_SEL_ to the drain. This configuration ensures that only the target device at the intersection experiences the voltage difference of V_SEL_, allowing conductance modulation through FN tunneling. Meanwhile, other devices on the same gate or drain lines experience only 1/2V_SEL_, which is insufficient to alter the conductance through FN tunneling. Figure 5c shows the programming results of the selected device using the half‐pulse scheme, where the +1/2V_SEL_ was set to –4.5 V for potentiation. The conductance of the selected device (Device 5) increased from 18.7 to 440.8 nS, while adjacent devices (Device 1–4) exhibited negligible changes in conductance, demonstrating that the half pulse scheme can successfully program the target device without interference. Our synaptic transistor with the ITO FG shows superior device performance compared with other FN tunneling‐based IGZO synaptic transistors in Table S1 (Supporting Information).

To verify the performance of the synaptic transistor in a neuromorphic system, we conducted SNN simulations using the MNIST and Fashion‐MNIST datasets,^[^ ^58^, ^59^ ^]^ as shown in Figure 5d. The network architecture consisted of three fully connected layers (256‐128‐10), and we employed offline learning that transfers pre‐trained weights to the synaptic devices for weight training.^[^ ^60^ ^]^ Although offline learning can offer high accuracy for complex datasets,^[^ ^61^ ^]^ the careful engineering of device‐to‐device variation and retention characteristics should be performed to ensure high accuracy because offline learning does not involve real‐time learning compensating the device variations within the SNN system.^[^ ^62^ ^]^ Figure 5e,f illustrates the SNN accuracy, considering device‐to‐device variation and retention characteristic, for the MNIST and Fashion‐MNIST datasets, respectively. High accuracies of 98.31% and 87.76% were achieved for the MNIST and Fashion‐MNIST datasets, respectively, even with device variation and weight degradation after 10^4^ s. Figure 5g represents the distribution of accuracies from 20 simulations that include variations and retention of devices. The lowest accuracies observed were 98.15% for MNIST and 85.47% for Fashion‐MNIST, indicating that the SNN system employing ITO FG synaptic transistors can maintain high accuracy with minimal performance degradation.

## 3 Conclusion

In summary, high‐performance synaptic transistors were fabricated using an ITO FG, which exhibited superior program/erase operations and retention compared to devices with IGZO FG. The enhanced performance is attributed to the ITO FG's higher conductivity and minimized chemical interactions with the ALD‐deposited Al₂O₃ TL, resulting in a smoother FG/TL interface and reduced defects. An 8 x 8 array using synaptic transistors was fabricated, achieving a 100% yield and successful programming without interference using a half‐pulse scheme. Furthermore, SNN simulations with MNIST and Fashion‐MNIST datasets demonstrated high accuracies of 98.31% and 87.76%, respectively, even when applying the device variations and retention. These results suggest that ITO FG synaptic transistors can be one of the promising candidates for neuromorphic computing applications.

## 4 Experimental Section

### Device Fabrication of Synaptic Transistors

A thermally grown SiO_2_/Si substrate was cleaned by acetone and IPA. The ITO gate layer (30 nm) was deposited by RF sputtering at room temperature (RT) with Ar plasma (70 W) and patterned by wet etching. Using the thermal ALD, the Al_2_O_3_ blocking layer (30 nm) was deposited at 150 °C using TMA and H_2_O as a precursor and oxidant, respectively. Next, IGZO (50 nm) or ITO (20 nm) floating gate was deposited by RF sputtering at RT with Ar plasma (100 W for IGZO and 70 W for ITO) and patterned by wet etching. The Al_2_O_3_ TL (9 nm) was deposited using ALD with the same process condition of the blocking layer. For the channel formation, 6 nm‐thick IGZO was deposited by RF sputtering at RT with Ar plasma (18 W) and patterned by wet etching. Subsequently, e‐beam evaporated Ti/Ni stack (40/20 nm) was patterned by a lift‐off process for the formation of source/drain electrodes. The Al_2_O_3_ passivation layer (10 nm) was deposited using ALD at 150 °C. Finally, the devices were annealed at 150 °C for 1 h in an air atmosphere. Detailed device specifications are provided in Figure S15 (Supporting Information).

### Characterization

All electrical measurements were conducted using a semiconductor parameter analyzer (Keithley 4200A‐SCS & 4225‐PMU) in the dark box at RT. Cross‐sectional images and EDS mapping were measured by TEM (Thermo Fisher, Themis Z). XPS (PHI, VersaProbe‐III) measurements were conducted using Al Kα (1486.6 eV) source with an Ar ion gun with an acceleration of 3 kV for depth profiling. REELS and UPS measurements were performed using XPS (Thermo Fisher, Nexsa). A He I source (21.22 eV) was used for the UPS measurement. The structural properties were investigated by GIXRD (PANalytical, X'pert Pro). The Tauc plots of films were obtained by an UV‐vis spectrometer (Hitachi, U‐2900).

## Conflict of Interest

The authors declare no conflict of interest.

## Supporting information



Supporting Information

## Data Availability

The data that support the findings of this study are available from the corresponding author upon reasonable request.
