# Supplementary material for: High‐Performance Synapse Arrays for Neuromorphic Computing via Floating Gate‐Engineered IGZO Synaptic Transistors
Source: Adv Sci (Weinh). 2025 Mar 20;12(21):2500568. doi: 10.1002/advs.202500568 (PMC12140291; doi:10.1002/advs.202500568)
Supplement: Supplementary file 1 — Supporting Information [file ADVS-12-2500568-s001.pdf]

ADVANCED SCIENCE
Open Access

Supporting Information

for *Adv. Sci.*, DOI 10.1002/advs.202500568

High-Performance Synapse Arrays for Neuromorphic Computing via Floating Gate-Engineered IGZO Synaptic Transistors

*Junhyeong Park, Yumin Yun, Sunyeol Bae, Yuseong Jang, Seungyoon Shin and Soo-Yeon Lee**

Supporting Information

High-Performance Synapse Arrays for Neuromorphic Computing via Floating Gate-Engineered IGZO Synaptic Transistors

*Junhyeong Park, Yumin Yun, Sunyeol Bae, Yuseong Jang, Seungyoon Shin, and Soo-Yeon Lee**

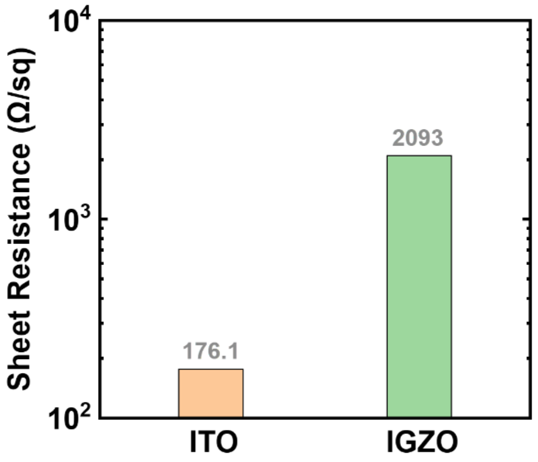

Figure S1. Sheet resistance of ITO and IGZO FGs measured using four-point probe measurements.

To measure the resistance of the floating gate (FG), Al_2O_3/ITO/Al_2O_3 and Al_2O_3/IGZO/Al_2O_3 samples were fabricated and annealed at 150 ℃ for 1 hour. Since the ITO and IGZO layers were covered with Al_2O_3, the Al_2O_3 layer were selectively etched using tetramethylammonium hydroxide (TMAH). Subsequently, the sheet resistance of ITO and IGZO layers was measured using a four-point probe.

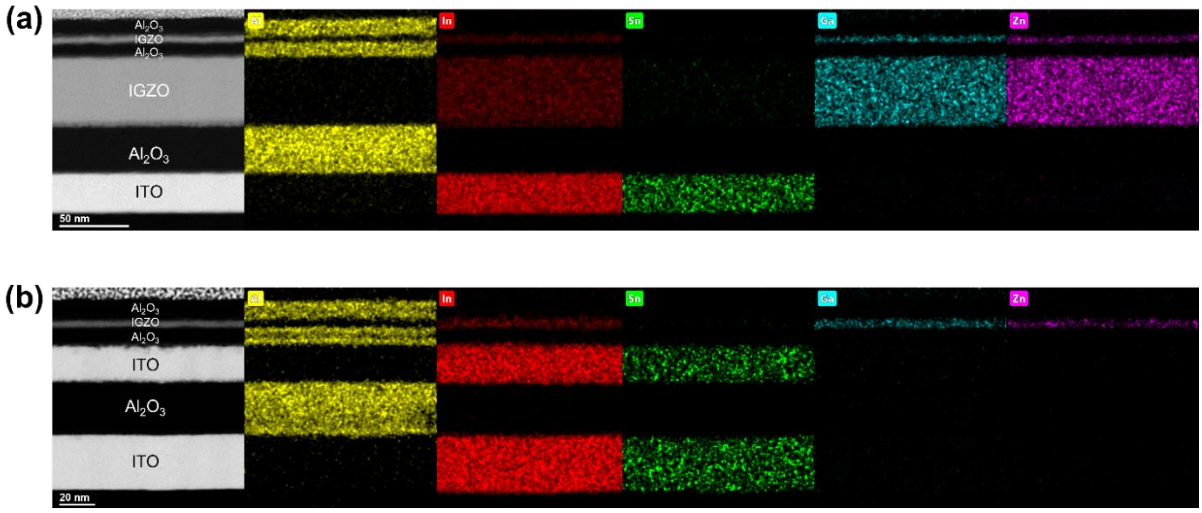

Figure S2. EDS elemental mapping images of the synaptic transistors with (a) IGZO and (b) ITO FGs.

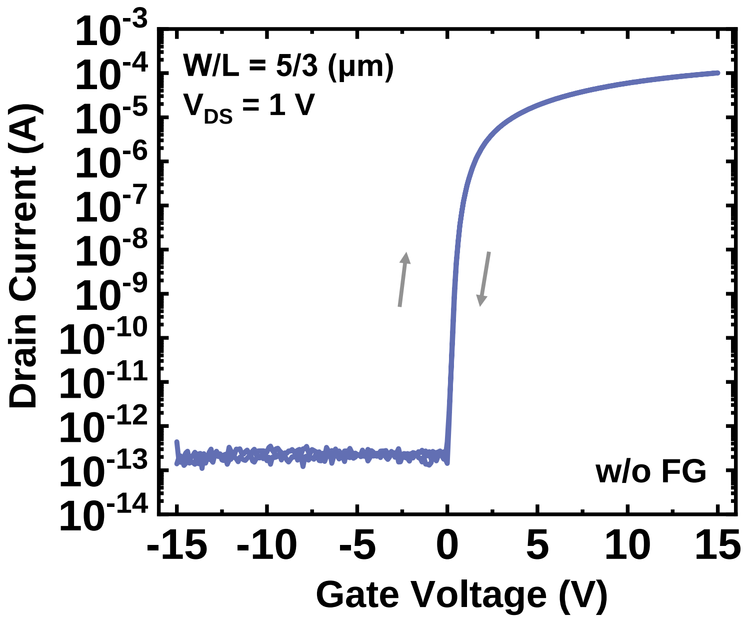

Figure S3. Transfer curve of the switching transistor without FG, measured under a gate double sweep from -15 V to 15 V.

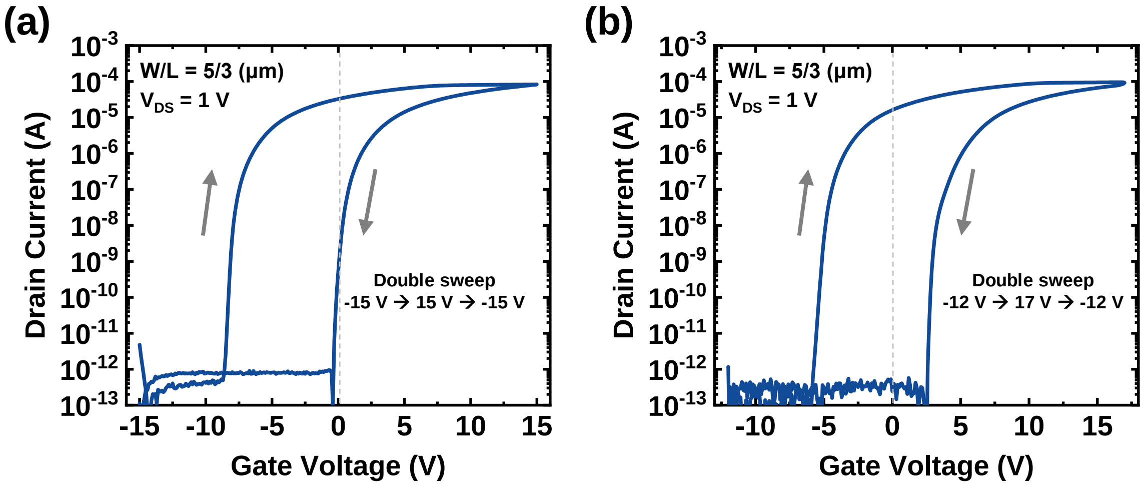

Figure S4. Transfer curves of synaptic transistors with ITO FG measured under sweep ranges of (a) -15 V to 15 V and (b) -12 V to 17 V.

When the transfer curve measurement is performed, the gate voltage sweep starts at a large negative voltage and then increases to a large positive voltage. Because a large voltage is applied at the beginning of the sweep, significant charge de-trapping (erase) occurs, resulting in a V_{TH} shift to strong depletion region. However, if the gate voltage increases to 17 V, the V_{TH} can increase to 3 V as shown in **Figure S4**b, representing the device can operate in enhancement mode.

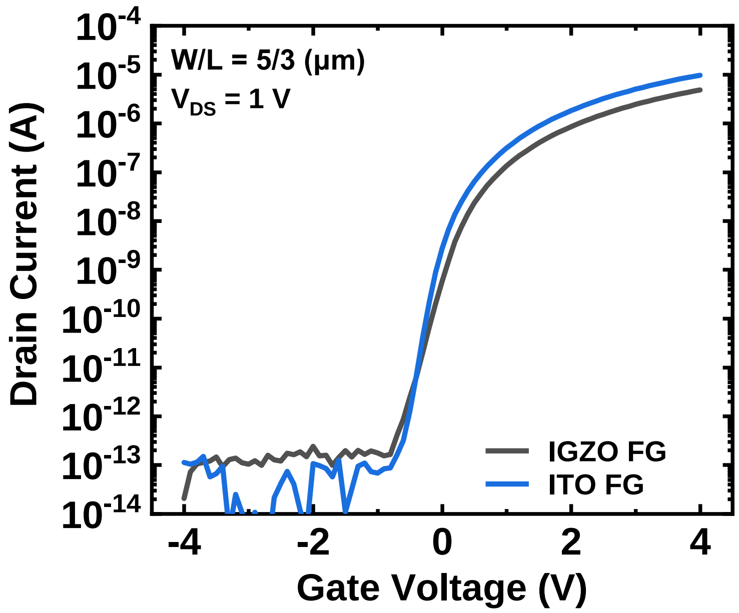

Figure S5. Transfer curves of pristine state of synaptic transistors. The devices exhibit similar initial V_{TH} of 0 V and 0.1 V for ITO FG and IGZO FG, respectively.

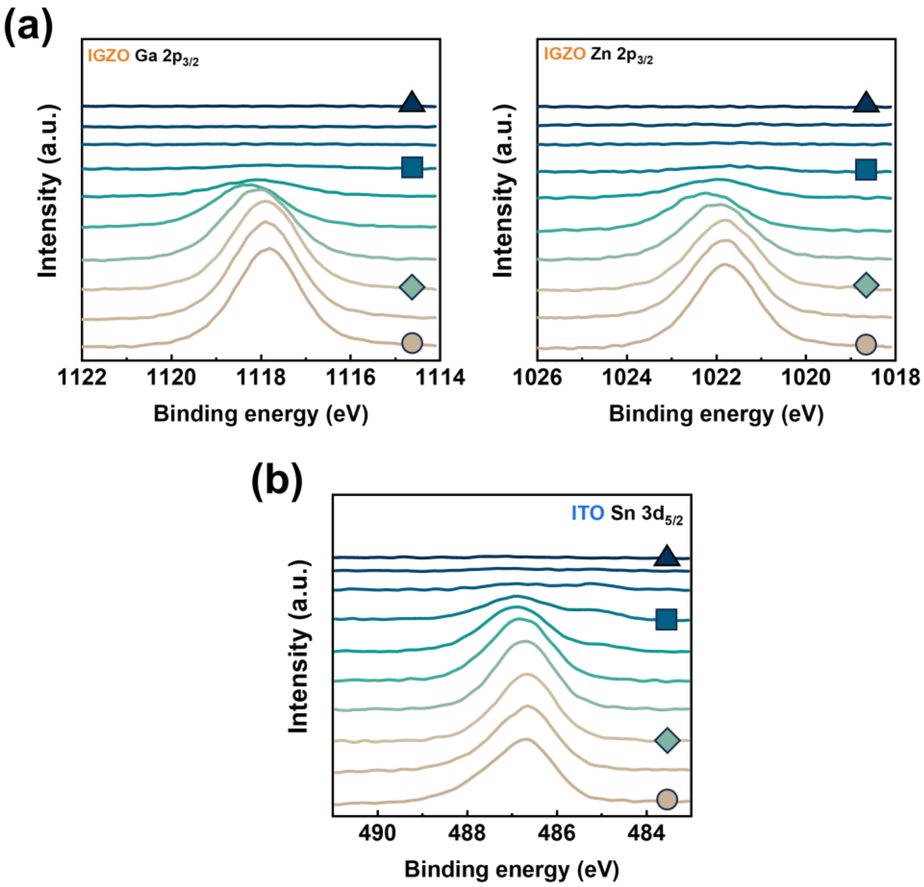

Figure S6. Depth-resolved XPS core level spectra of (a) Ga $2p_{3/2}$ and Zn $2p_{3/2}$ for Al_2O_3/IGZO, and (b) Sn $3d_{5/2}$ for Al_2O_3/ITO.

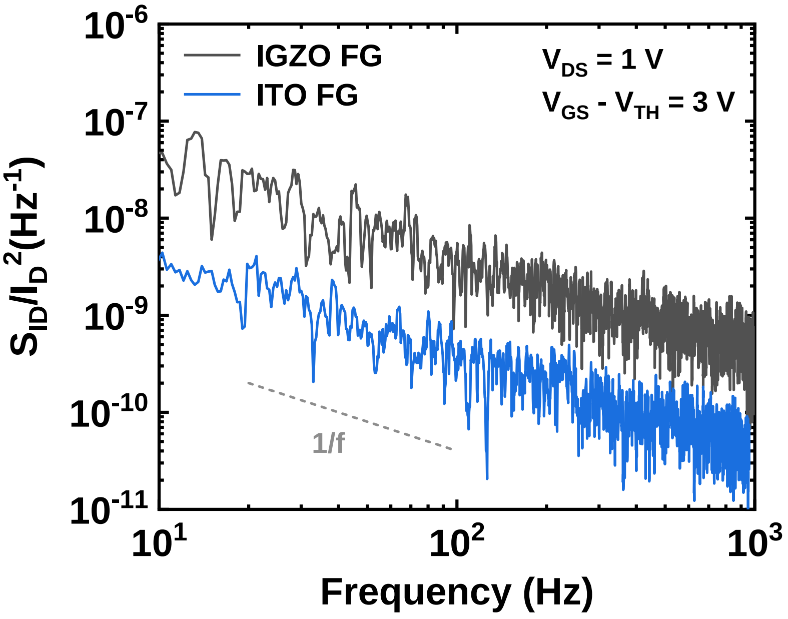

Figure S7. Normalized noise spectral density (S_{ID}/I_D^2) for the synaptic transistors with IGZO and ITO FGs.

For the 1/f noise measurement, the drain current sampling of synaptic transistors was carried out using the Keithley 4200A-SCS and 4225-PMU, and the normalized noise spectral density was calculated by using a fast Fourier transform. The results show that the IGZO FG exhibits higher noise compared to the ITO FG, which is attributed to a larger defect near the interface. This measurement result is consistent with the results of the poor interface of IGZO FG confirmed by using XPS and TEM.

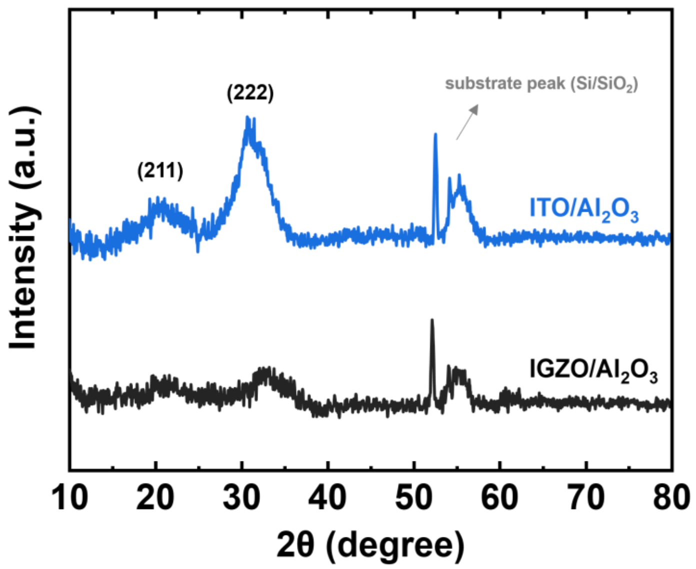

Figure S8. GIXRD patterns of ITO/Al_2O_3 and IGZO/Al_2O_3 stacks.

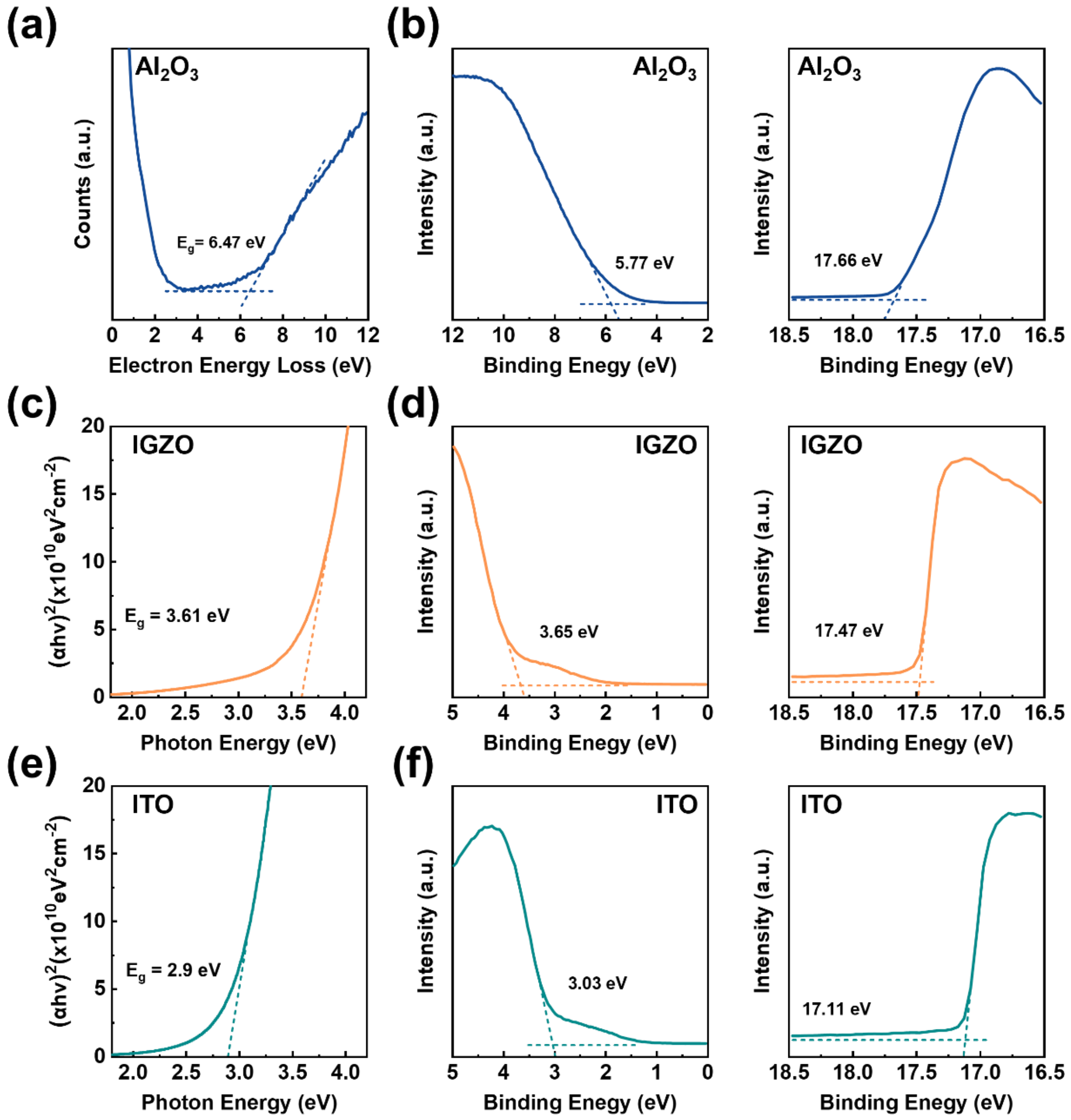

	Al_2O_3	IGZO	ITO
E_c	2.86 eV	3.79 eV	4.24 eV
E_f	3.56 eV	3.75 eV	4.11 eV
E_v	9.33 eV	7.4 eV	7.14 eV
E_g	6.47 eV	3.61 eV	2.9 eV

Figure S9. (a) REELS and (b) UPS spectra of the Al_2O_3 dielectric layer. (c) UV-vis and (d) UPS spectra of the IGZO FG layer. (e) UV-vis and (f) UPS spectra of the ITO FG layer. The energy levels of layers were calculated based on the measurement results.

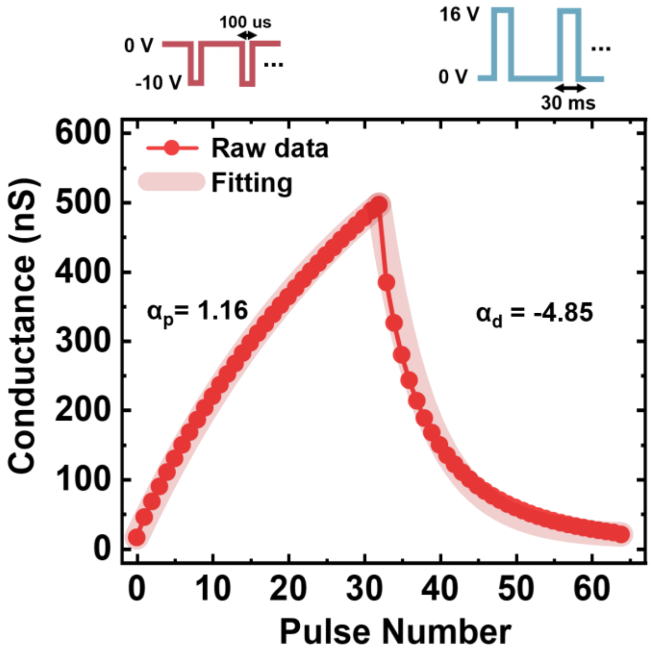

Figure S10. LTP/LTD measurement of the synaptic transistor using the identical pulse scheme for both potentiation and depression.

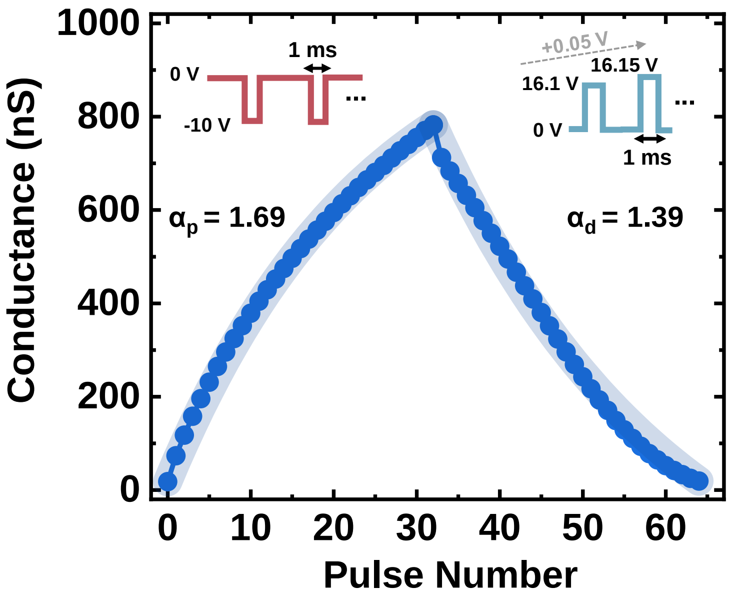

Figure S11. LTP/LTD measurements of the synaptic transistor using the same voltage pulse width (1 ms) for both LTP and LTD.

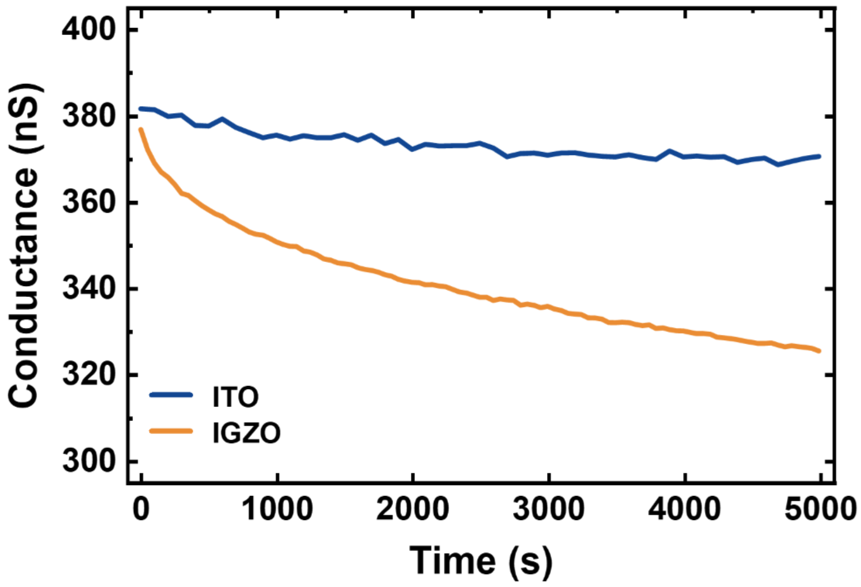

Figure S12. Retention comparison of ITO and IGZO FGs for $5x10^3$ s.

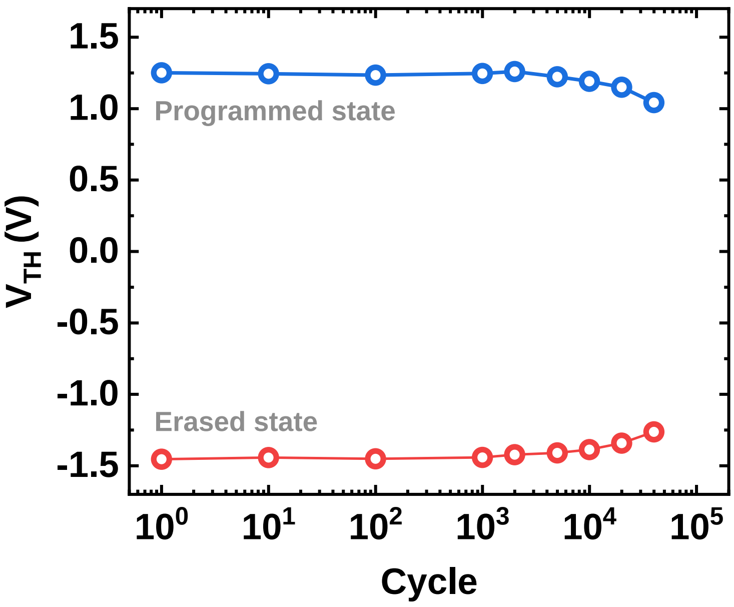

Figure S13. Endurance result of repetitive program and erase operations using the synaptic transistor with ITO FG. Program and erase operations were performed with voltage pulses of 18 V (5 ms) and -12.5 V (100 μs), respectively.

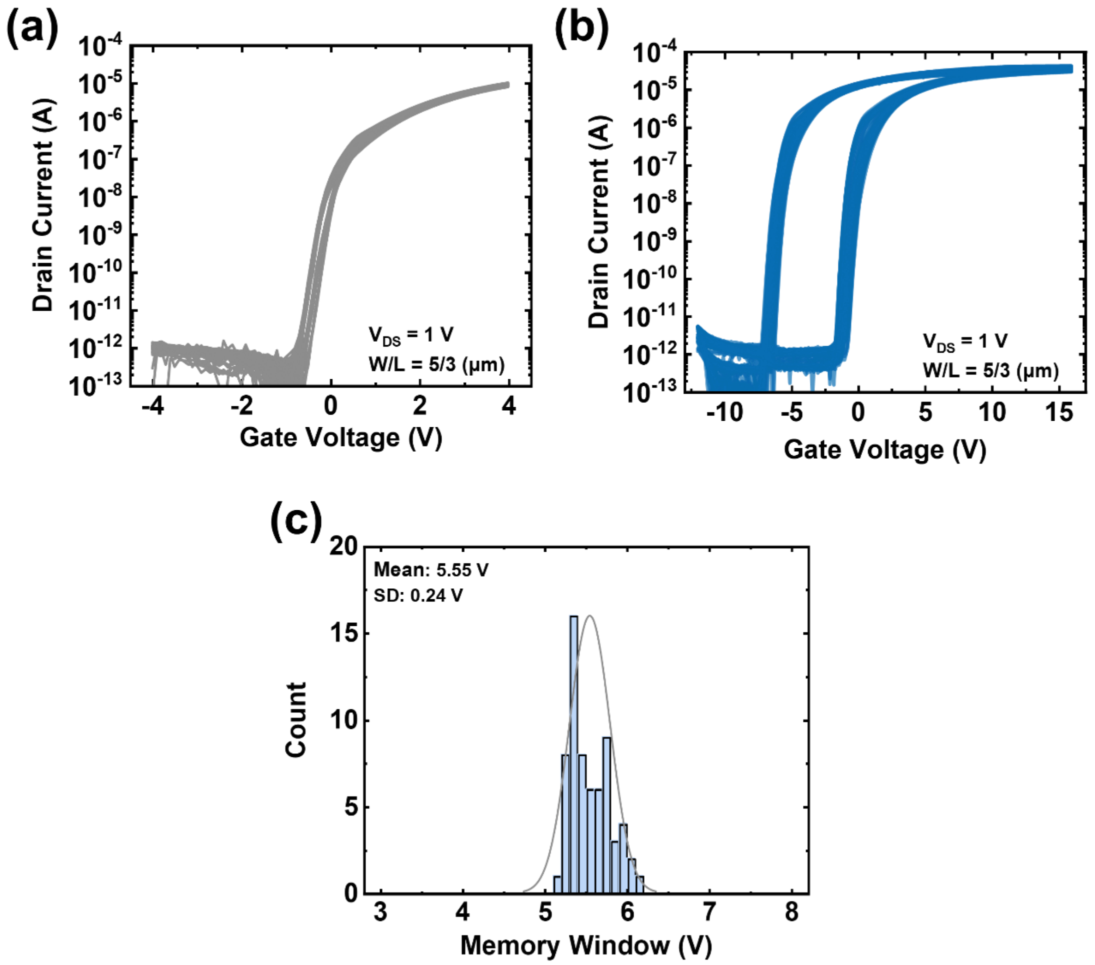

Figure S14. (a) Transfer curves of 64 pristine devices, exhibiting the uniform initial V_{TH}. (b) Transfer curves of 64 devices with a yield of 100%, measured under a gate double sweep from -12 V to 15 V, and (c) distribution of the memory window of the 64 devices.

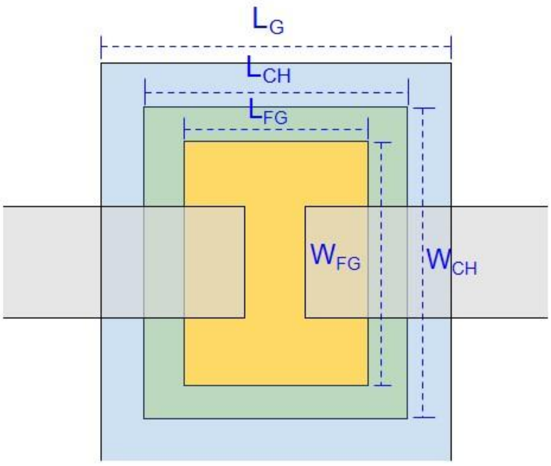

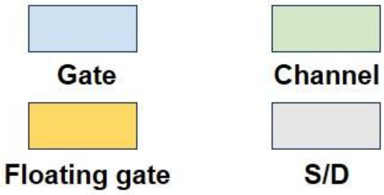

Device parameters			
W/L	5/3 μm	L_G	17 μm
W_{CH}	14 μm	L_{CH}	13 μm
W_{FG}	11 μm	L_{FG}	9 μm

Figure S15. Device specifications of fabricated synaptic transistors.

Table S1. Comparison of recently reported FN tunneling-based IGZO synaptic transistors.

Device	Ref. [1]	Ref. [2]	Ref. [3]	This work
Gate stack	Al_2O_3/IGZO/Al_2O_3	HfO_2+Al_2O_3/ Si_3N_4/Al_2O_3	SiO_2/TaO_x/Al_2O_3	Al_2O_3/ITO/Al_2O_3
Array fabrication	-	-	-	8x8
Process temperature	≤200 °C	≤300 °C	≤450 °C	≤150 °C
Nonlinearity[a)]	-0.03/-0.47	-	1.99/0.44[b)]	0.99/1.08
Potentiation pulse	-20 V (100 μs)	14 V (1 ms)	-15 V (10 μs)	-10 V (100 μs)
Depression pulse	6 ~ 20 V (5 ms)	13 V (1 ms)	5.7 V (10 μs)	14.8 ~ 16.35 V (40 ms)
Retention	60 s (32 states)	10^4 s (11 states)	10^4 s (1 state)	10^4 s (32 states)
Endurance (Program/Erase)	1000 cycles	-	-	40000 cycles
Endurance (LTP/LTD)	32000 pulses	-	2000 pulses	25600 pulses
Network accuracy	98.08% (MNIST)	83.9% (MNIST)	95.01% (MNIST)	98.31% (MNIST) 87.76% (Fashion-MNIST)

[a)]ideal value is 0; [b)] Nonlinearity is extracted using different fitting equations;

[1] J. Park, Y. Jang, J. Lee, S. An, J. Mok, S.-Y. Lee, *Adv. Electron. Mater.* **2023**, 9, 2201306.
[2] E. Park, S. Jang, G. Noh, Y. Jo, D. K. Lee, I. S. Kim, H.-C. Song, S. Kim, J. Y. Kwak, *Nano Lett.* **2023**, 23, 9626.
[3] J. Jang, S. Park, D. Kim, S. Kim, *Sens. Actuators A: Phys.* **2024**, 376, 115641.
